# What We Know about *Euterpe* Genus and Neuroprotection: A Scoping Review

**DOI:** 10.3390/nu15143189

**Published:** 2023-07-19

**Authors:** Ilano Oliveira Da Silva, Maria Elena Crespo-Lopez, Marcus Augusto-Oliveira, Gabriela de Paula Arrifano, Natália Raphaela Ramos-Nunes, Elielton Barreto Gomes, Felipe Rodolfo Pereira da Silva, Aline Andrade de Sousa, Alessandro Luiz Araújo Bentes Leal, Helane Conceição Damasceno, Ana Carolina Alves de Oliveira, José Rogério Souza-Monteiro

**Affiliations:** 1 Medicine College, Altamira Campus, Federal University of Pará (UFPA), Altamira 68372-040, PA, Brazil; ilanooliveiradasilva@gmail.com (I.O.D.S.); alinebio02@gmail.com (A.A.d.S.); alessandroluisaraujo@gmail.com (A.L.A.B.L.); helanehd@hotmail.com (H.C.D.); anacarolina@ufpa.br (A.C.A.d.O.); 2 Laboratory of Molecular Pharmacology, Institute of Biological Sciences, Federal University of Pará, Belém 66075-110, PA, Brazil; ecrespo@ufpa.br (M.E.C.-L.); marcusadeoliveira@outlook.com (M.A.-O.); arrifanogabriela@gmail.com (G.d.P.A.)

**Keywords:** Amazon, neuroprotection, *Euterpe*, *Euterpe oleracea*, *Euterpe edulis*, *Euterpe precatoria*, açaí, juçara, CNS, brain

## Abstract

The *Euterpe* genus (mainly *Euterpe oleracea* Martius, *Euterpe precatoria* Martius, and *Euterpe edulis* Martius) has recently gained commercial and scientific notoriety due to the high nutritional value of its fruits, which are rich in polyphenols (phenolic acids and anthocyanins) and have potent antioxidant activity. These characteristics have contributed to the increased number of neuropharmacological evaluations of the three species over the last 10 years, especially açaí of the species *Euterpe oleracea* Martius. The fruits of the three species exert neuroprotective effects through the modulation of inflammatory and oxidative pathways and other mechanisms, including the inhibition of the mTOR pathway and protection of the blood–brain barrier, all of them intimately involved in several neuropathologies. Thus, a better understanding of the neuropharmacological properties of these three species may open new paths for the development of therapeutic tools aimed at preventing and treating a variety of neurological conditions.

## 1. Introduction

Diets rich in fruits and vegetables are beneficial for the central nervous system (CNS). Such benefits are attributed to the richness and diversity of micronutrients, macronutrients, and phenolic compounds, which are important for brain homeostasis and neuroprotection [1,2,3,4].

There has recently been a significant increase in scientific production aimed both at identifying the phytochemical composition and at evaluating the pharmacological effects of the fruits and seeds of the species *Euterpe oleracea* Martius (EO), *Euterpe precatoria* Martius (EP), and *Euterpe edulis* Martius (EE) through preclinical and clinical studies [5,6,7,8,9,10,11,12].

Although *Euterpe* species are not endemic to Brazil, they are found in different states and phytogeographic domains [13,14,15]. EO palm is found in Venezuela, Guyana, and the Brazilian Amazon (in the estuary of the Amazon River), in the northern and northeastern states, and is popularly known as açaí-do-Pará [13,16,17,18]. Like EO, the occurrence of EP palm has been confirmed in northern Brazil and is popularly known as açaí-do-Amazonas or açaí-da-mata. This palm can also be found in countries such as Equator, Peru, and Bolivia [14,16,18]. The fruits derived from these two species are known as açaí, a black-purple fruit. EO and EP species are scientifically and commercially recognized for the açaí pulp or juice produced from their fruits [16,19,20,21]. In this work, açaí derived from EO will be identified as AEO, and açaí pulp from EP will be designated as AEP.

EE species is a palm known in Brazil as juçara, palmiteiro, or palmito-juçara [8,10]. Unlike the previous species that are recognized for their fruits, EE palm is recognized mainly for the heart of palm; however, the fruits of EE (which in this work will be called juçara fruit or JF) are similar to those of AEO and AEP, being spherical purple fruits, with similar sensory characteristics and high antioxidant activity. EE has no confirmed occurrence in the Amazon, with the Cerrado and the Atlantic Forest as its characteristic phytogeographic domains, with confirmed occurrence in the northeastern, midwestern, southeastern, and southern areas of Brazil [10,15,22,23,24].

Considering that the fruits of these three species are exotic with proven beneficial health effects due to their nutritional properties and the presence of a wide variety of phenolic compounds, these fruits received the status of “superfruits”, a term representing a marketing strategy that has contributed to increasing their popularity and high consumption in Brazil and worldwide [5,25,26,27,28]. The fruits of these three species have a similar polyphenolic profile, and the presence of these bioactive substances has aroused scientific interest [7,10,18,23,27,29,30]. The particular scientific interest in these natural compounds is related to the neuroprotective activity of many flavonoid and non-flavonoid molecules, which exert effects through a combination of multiple mechanisms of action that protect the CNS from neuroinflammation and damage induced by oxidative stress [7,10,18,25,31].

Oxidative stress is the imbalance between the generation of reactive oxygen (ROS) and nitrogen (RNS) species and the antioxidant defense capacity. Faced with the inefficiency of antioxidant defense systems, ROS (e.g., superoxide, hydroxyl, and hydrogen peroxide) and RNS (e.g., nitric oxide and peroxynitrite) might promote the oxidation of biomolecules (proteins, carbohydrates, lipids, and nucleic acids), homeostatic imbalance, and tissue damage [32,33,34,35]. Regarding the CNS, it is important to highlight that the brain is the target organ of oxidative damage and that oxidative stress is involved in the pathogenesis of many disorders affecting the nervous system due to the induction of neuronal death, neuroinflammation, and neurodegeneration [33,36,37,38].

Neuroinflammation is also a hallmark of the pathogenesis of some CNS diseases, characterized by the involvement of microcirculation, cellular components (e.g., microglial cells and astrocytes), and inflammatory mediators [39,40,41]. Oxidative stress can induce a neuroinflammatory state through the activation of signaling cascades leading to glial reactivity. These cells contribute to oxidative stress and neuroinflammation through overproduction and release of cytokines and oxigen species, which may represent a risk if maintained in long-term [42]. This intimate relationship raises questions about the interdependence between oxidative stress and inflammatory response and the need to develop new therapies aimed at both preventing and mitigating pathological outcomes [43].

Oxidative stress and inflammation are involved in the pathogenesis of several diseases that affect the CNS (e.g., epilepsy and depression) [44,45], and even in MeHg-induced neurotoxicity [46,47]. Scientific evidence demonstrates the rich phytochemical composition and the potent antioxidant and anti-inflammatory effects of the fruits of EO, EP, and EE species, and there is no synthesis review providing all the compiled knowledge on the neuroprotective properties of fruits and other plant organs of these three species of the genus *Euterpe*. Thus, it is important to identify and describe the evidence available so far on such properties. In this sense, this article sought to gather information (mechanistic and methodological) to generate a broad, consistent, and understandable overview of the neuroprotective effects of açaí and juçara.

## 2. Materials and Methods

**Figure 1:**
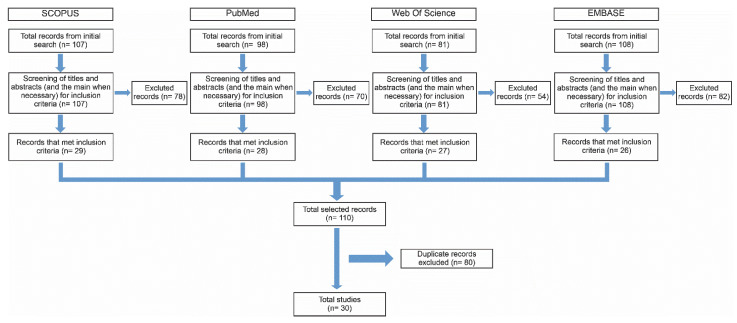
Flowchart of the search strategy performed in this scoping review.

This scoping review was performed according to the PRISMA guidelines for scoping reviews [48]. The systematic search was performed in the PubMed/Medline, SCOPUS, EMBASE, and Web of Science databases on 31 January 2023, without restriction of language or year of publication. The terms used for the bibliographic search in the databases were as follows: Euterpe AND neuroprotective, açaí AND brain, Euterpe AND brain, Euterpe AND astrocytes, Euterpe AND microglia, Euterpe AND neuroprotection, açaí AND Nrf-2, açaí AND neurodegenerative disorders, and Euterpe AND neurodegenerative disorders. These terms were searched in the title, abstract, and keywords fields of the referred databases. The inclusion criteria were original article, experimental studies (*in vivo* and *in vitro*), and studies that evaluated the pharmacological effects of *Euterpe* species (*E. oleracea*, *E. precatoria*, and *E. edulis*) in the CNS (tissue and/or cells-neurons and glia). Were excluded non-original and/or non-experimental articles, book chapters, summaries, reviews, patents, conference abstract, meeting abstract, editorial and articles that did not investigate the neuropharmacological action of the *Euterpe* species in *in vivo* or *in vitro* studies. Duplicate studies were excluded (Figure 1). The articles were independently revised by two different authors. When in doubt, the full article was consulted. The extracted data included year of publication, part of the plants used in *in vitro* or *in vivo* assays and cell lines intended for neuropharmacological evaluation.

## 3. Results and Discussion

### 3.1. Features of Selected Studies

**Figure 2:**
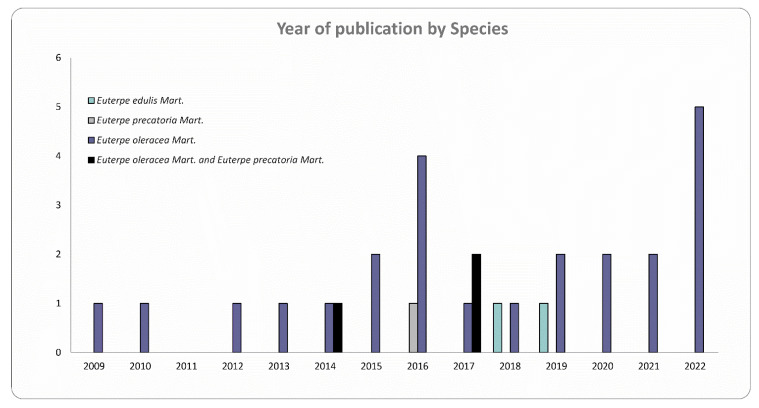
Number of articles per year analyzing the effects of each species of the *Euterpe* genus.

A total of 394 articles were found, but only 30 studies were included based on the inclusion/exclusion criteria (Figure 1). Studies evaluating the pharmacological effects of AEO, AEP, and JF on the CNS are relatively recent, with the first evidence published in 2009 demonstrating, through an *in vitro* study, that AEO pulp reduced the oxidative damage induced by H_2_O_2_ in proteins and lipids of the cerebral cortex, hippocampus, and cerebellum *in vitro* [49]. Subsequently, from 2010 to 2015, only seven articles were published, six of which had the main objective of evaluating the neuropharmacological actions of AEO. Interestingly, since 2016, there has been a considerable increase in the number of studies evaluating the pharmacological actions of the three *Euterpe* species on the CNS. In 2022, five articles were published focusing on the assessment of AEO in the CNS (Figure 2).

In addition to the number of studies, Figure 2 shows the differences between the number of publications among the three *Euterpe* species. Only one study analyzed the pharmacological effects of AEP on the CNS, two studies evaluated the effects of JF, three studies (10% of selected items) investigated both AEO and AEP, and a total of 24 studies (80% of the selected items) evaluated the fruits and seeds of EO species. Therefore, EO is clearly the most frequently studied species at the present time.

Neuropharmacological studies with AEP and JF are recent and scarce, which indicates the great inequality of studies published in relation to the three different species of *Euterpe* evaluated in this study. A possible explanation for the greater number of scientific studies with AEO is related to the increase in the consumption, production, and market of AEO, both in Brazil and internationally [28,50], which favors the access of populations to açaí pulp, as well as products of AEO, such as energy drinks. This wide international access to pulp AEO drew the attention of the scientific community because it is a fruit rich in bioactive compounds with high antioxidant and anti-inflammatory activities; that is, the “scientific popularity” is not only associated with its nutritional properties but mainly to a variety of biological activities and their potential beneficial health effects [5,18,29,51,52]. The imbalance between the number of studies with AEP and JF compared to studies with AEO indicates that there is a gap in the literature and a much to be explored by studies that can evaluate the biological activities of fruits and other plant parts of the EE and EP species in the CNS and even in other biological systems.

As AEO, AEP and JF also play neuroprotective roles in experimental models (Table 1). The greater number of studies with AEO is well justified, but according to the data in Table 1, there is evidence to suggest that AEP and JF deserve greater attention from the scientific community, as the fruits of these plant species have a rich phytochemical composition and particular characteristics related to neuroprotection. When compared to AEO, AEP is richer in phenolic compounds and has superior antioxidant capacity, while JF has a higher total phenolic content than that found in AEP and AEO [23,53,54]. These data are relevant and would justify greater attention from the Brazilian and international consumer market regarding EP and EE fruits, with sustainable exploitation, in addition to a greater density of scientific studies.

**Figure 3:**
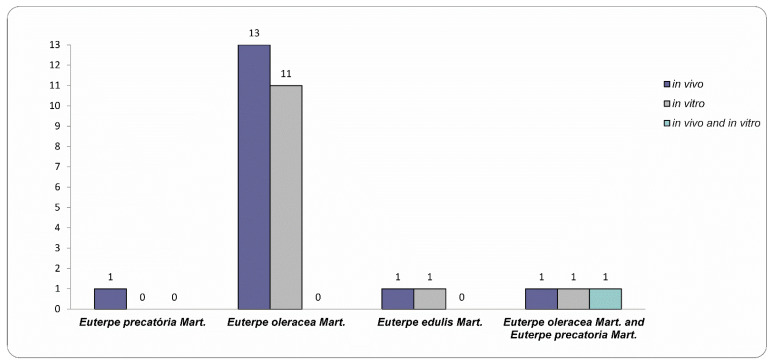
Number of *in vivo* and *in vitro* studies according to *Euterpe* species.

The neuropharmacological evaluation of the three *Euterpe* species was carried out through preclinical studies (*in vivo* and *in vitro* assays) (Figure 3), assays that are crucial for the development of new drugs [80]. Based on the analysis of the selected articles, we summarized the experimental models and the main neuroprotective actions of the AEO, AEP, and JF (Table 1). Clinical studies with AEO and JF published so far do not assess the effects of these species on the CNS in humans; however, these studies in humans demonstrate that the fruits of these species are able to modulate the inflammatory response related to overweight and obesity and to improve the antioxidant defense. The neuroprotective effects described in Table 1 and the protective effects already demonstrated by clinical studies (modulation of the inflammatory response, improved HDL-c levels, and antioxidant defense) are relevant to the point of justifying investments in clinical studies directed at the CNS [9,67,81,82].

Most of the selected studies (26 studies) were performed by using the fruits, and only three studies were performed with seeds. Among the selected studies, only the study by Yildirim et al. (2020) does not indicate which part of the plant was used in their experiments. The frequent use of the fruits in pharmacological evaluations is possibly justified by human consumption of the fruit in the form of açaí juice (AEO, AEP) and juçara juice [8,18,52], besides the wide availability of data on the phytochemical composition of the fruits [5,7,10,18,29]. Considering these aspects, there is a “trend” of carrying out studies about the fruits, which provides a better association of the results concerning human health.

However, an important fact to be considered in future studies with the three *Euterpe* species is the possibility of using other organs besides the fruit, since traditional Amazonian populations use organs such as the root and seed of EP and EO in folk medicine to treat clinical conditions including malaria [83,84], urinary tract infection, diarrhea, intestinal infection [85,86,87], verminosis [88], hemorrhoids, and varicose veins [89]. Data from these ethnopharmacological studies are strengthened by other scientific studies showing that EO seeds are rich in proanthocyanidins, compounds with several beneficial effects, including anti-inflammatory and antioxidant actions. These pharmacological actions are important and may justify the use of seeds in future preclinical studies on the nervous system [90,91,92,93]. In addition to seeds, roots, leaflets, flowers, and spikes of EO can also be targets for future neuropharmacological studies. Brunschwig et al. (2016) evaluated the phytochemical composition and antioxidant activity of EO roots and leaflets and demonstrated that these organs have antioxidant activity and are rich in compounds such as hydroxycinnamic acids and flavones, compounds with anti-inflammatory and antioxidant activity [94,95]. The flowers and spikes of EO were able to inhibit the production of NO and the expression of inducible nitric oxide synthase (iNOS) in RAW 264.7 cells; however, as they are monocyte/macrophage-like cells, similar results have already been observed in neuropharmacological evaluations with the fruits of EO and EP in the BV-2 microglia cell line (see Table 1) [77,79,96]. In general, these results are representative and strengthen the idea that future research should be carried out with the aim of studying the phytochemical composition and subsequently the neuropharmacological activity of other plant organs of the EO, EP, and EE species, in addition to the fruits.

### 3.2. Experimental Models to Study Neuroprotection

Regarding the types of preclinical research selected, 13 studies performed *in vitro* assays, 16 studies were conducted through *in vivo* assays, and only one study performed both *in vitro* and *in vivo* assays simultaneously (Figure 3).

**Figure 4:**
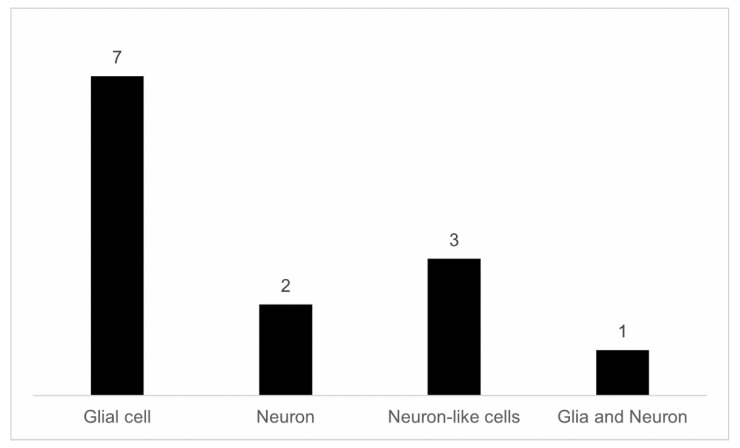
Number of studies and cell types used in *in vitro* studies.

Different from *in vivo* studies, *in vitro* experiments allow the isolation of the effects on one specific type of neural cell (neurons and glia). In this review, of the thirteen *in vitro* studies selected, only three studies were conducted using primary cultures (culture of neurons and astrocytes), while ten studies were performed using cell lines, which were as follows: microglia (EOC 13.31 cell line, BV-2), astrocytes (DI TNC1), neuronal-like cells (SHSY5Y), hippocampal neurons (HT22) and rat phaeochromocytoma cells (PC12). Primary cell cultures derive from the isolation of cells directly from the host tissue and have a finite lifespan and physiological characteristics similar to those of cells *in vivo*, whereas cell lines derived from subcultures of primary cells have a longer lifespan than primary cells and longer growth capacity. The likely explanations for the greater number of studies with secondary cell culture are the easy cultivation, the lower risk of contamination as compared to primary cultures and the challenge of performing the cultivation of neuronal cells, which once mature are not capable of undergoing cell division [30]. Another important aspect to be highlighted in relation to the selected *in vitro* studies is that they were performed in 2D cell cultures, with predominance of the use of microglia cells, astrocytes and scarcity of studies with neurons (Figure 4). As in *in vivo* studies, EO continued to be the main plant species studied and *in vitro* research that sought to evaluate the neuropharmacological actions of EP and EE remains scarce.

Interestingly, a question to be asked in light of these characteristics is the following: Why did the vast majority of studies use glial cells? These cells play a key role in neuroinflammation, oxidative stress, and the recycling of neurotransmitters. From the analysis of the selected articles, one can observe that AEO and AEP were able to reduce the inflammatory response (an effect observed mainly in cultures of microglial cell lines—EOC 13.31 and BV-2) and regulate the antioxidant response and even the GABA uptake in astrocytes [73,74,76,77,79]. It is important to highlight that microglia and astrocytes exert functions beyond neuroinflammation and oxidative stress; e.g., together, these cells participate in the regulation of neuronal activity and are components of the neurovascular unit [97]. Microglial functions reach further than CNS immunosurveillance and defense, orchestrating, together with other cells, brain homeostasis, adult neurogenesis, and synaptic plasticity, strongly influencing animal cognition and behavior [98,99]. Similarly, astrocytes play crucial roles in this homeostasis, including synaptic formation, maintenance and elimination, maintenance of the blood–brain barrier, and recycling of neurotransmitters, to name a few [100]. Considering the modulatory effects that AEO and AEP exert on these glial cells, it would be interesting if further studies could verify whether açaí (from EO and EP species) is capable of modulating homeostatic functions of microglia and astrocytes, such as neurogenesis.

When analyzing the experimental models and the main results of the *in vivo* studies selected in this scoping review (see Table 1), one can observe great diversity and heterogeneity between the experimental models used to evaluate the neuroprotective effects of the three *Euterpe* species. In these studies, models were used that mimic clinical conditions such as seizures, depressive behavior, anxiety, and hepatic encephalopathy, as well as a model of intoxication by the neurotoxicant MeHg—i.e., despite the different experimental models (*in vivo* and *in vitro*) used in the studies selected, the main conclusions observed refer to neuroprotection.

Among the neuroprotective effects of açaí from EO, one is particularly interesting, as it does not refer to the prevention of a disease that affects the CNS but rather to the prevention of the neurotoxicity induced by MeHg through the reduction of the malondialdehyde (MDA) and nitrite levels in the brain; that is, the antioxidant property of AEO was responsible for the neuroprotective effect [46]. This is a very expressive result for vulnerable populations (e.g., riverine inhabitants) of the Amazon, where human exposure to MeHg (organic compound of mercury) is an important public health problem associated with the intake of contaminated fish by riverine populations living in areas of artisanal and small-scale gold mining (ASGM) [101,102,103,104]. Considering that the antioxidant property of AEO was responsible for the neuroprotective effect against MeHg intoxication and that AEO is easily obtained and consumed regularly by these populations, it is possible to suggest that açaí is an excellent option to protect Amazonian riverine populations exposed to MeHg.

Although the antioxidant property of EO is well established [7,105,106], it was suggested that treatment with the EO seed extract could be pro-oxidant in a model of cancer [62], but methodological issues (such as the lack of a group treated only with the extract) prevent these results from being conclusive. All other studies presented in Table 1 demonstrated that the fruits and seeds of EO protected the CNS against oxidative stress by reducing biochemical parameters associated with both lipid peroxidation and nitric oxide production (malondialdehyde and nitrite levels) [46,55], increasing the activity of antioxidant enzymes (catalase, superoxide dismutase, and heme oxygenase-1) [61,73] and increasing the expression of Nrf2 (critical element in antioxidant defense) [51].

**Figure 5:**
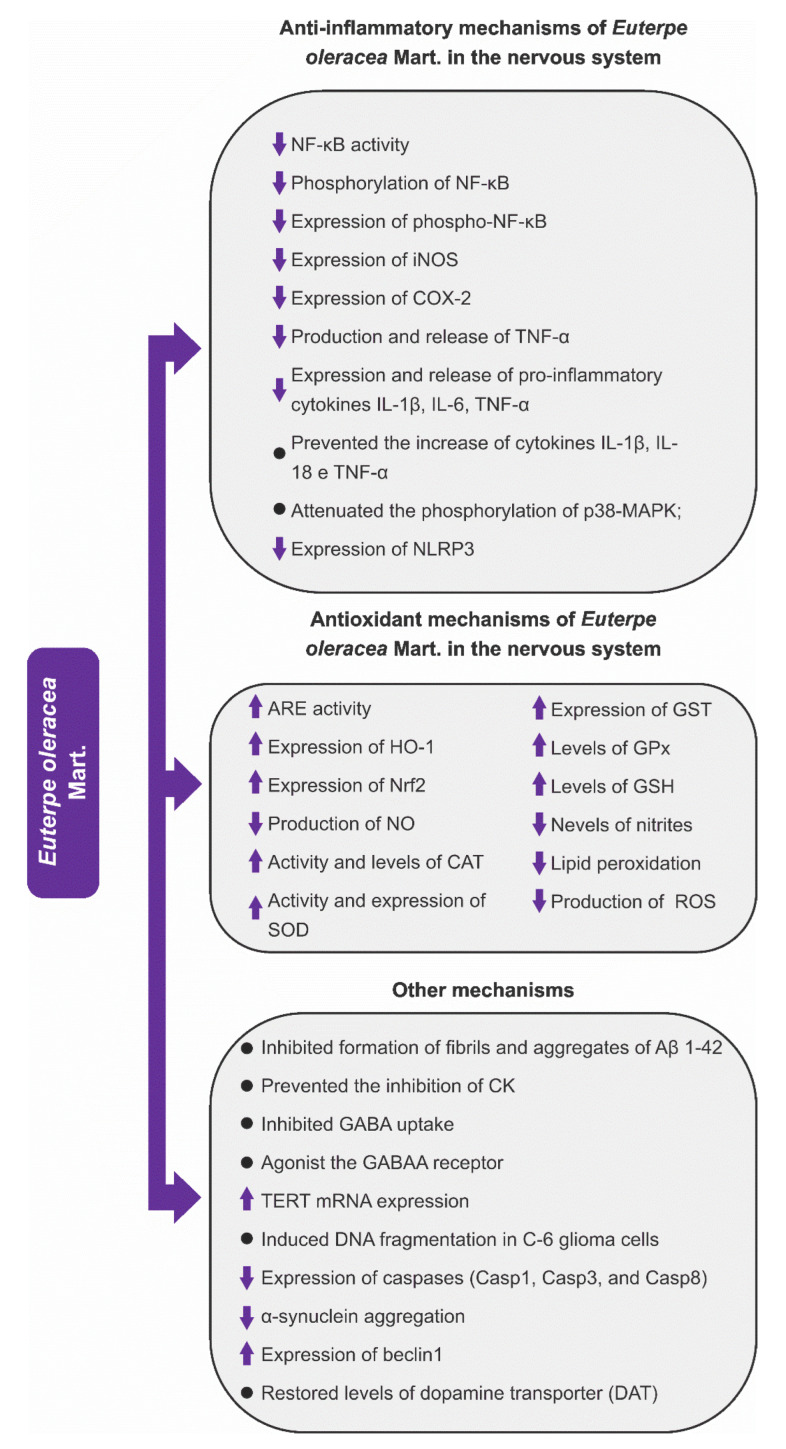
*Euterpe oleracea* Mart.—molecular mechanisms of neuroprotection. Abbreviations: NF-κB: nuclear factor κB; iNOS: inducible nitric oxide synthase; COX-2: cyclooxygenase-2; TNF-α: tumor necrosis factor-α; IL-1β: interleukin-1β; IL-6: interleukin-6; IL-18: interleukin-18; p38-MAPK: p38 mitogen-activated protein kinase; NLRP3: nod-like receptor pyrin containing 3; CK: creatine kinase; TERT: telomerase reverse transcriptase; ARE: Antioxidant Response Element; HO-1: heme oxygenase-1; Nrf2: Nuclear factor erythroid 2-related factor 2; NO: Nitric oxide; CAT: catalase; SOD: superoxide dismutase; GST: glutathione S-transferase; GPx: glutathione peroxidase; GSH: reduced glutathione; ROS: Reactive Oxygen Species.

**Figure 6:**
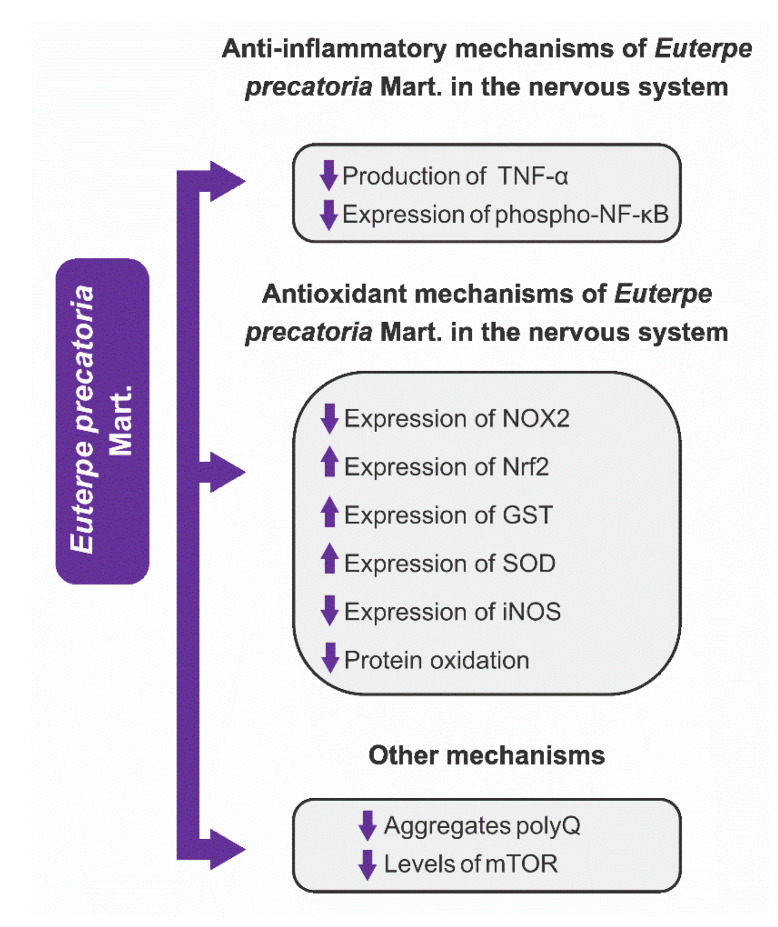
*Euterpe precatoria* Mart.—molecular mechanisms of neuroprotection. Abbreviations: TNF-α: tumor necrosis factor-α; NF-κB: nuclear factor κB; polyQ: polyglutamine; mTOR: mammalian target of rapamycin; NOX2: NADPH-oxidoreductase-2; Nrf2: NF-E2-related factor 2; GST: glutathione S-transferase; SOD: superoxide dismutase; iNOS: inducible nitric oxide synthase.

**Figure 7:**
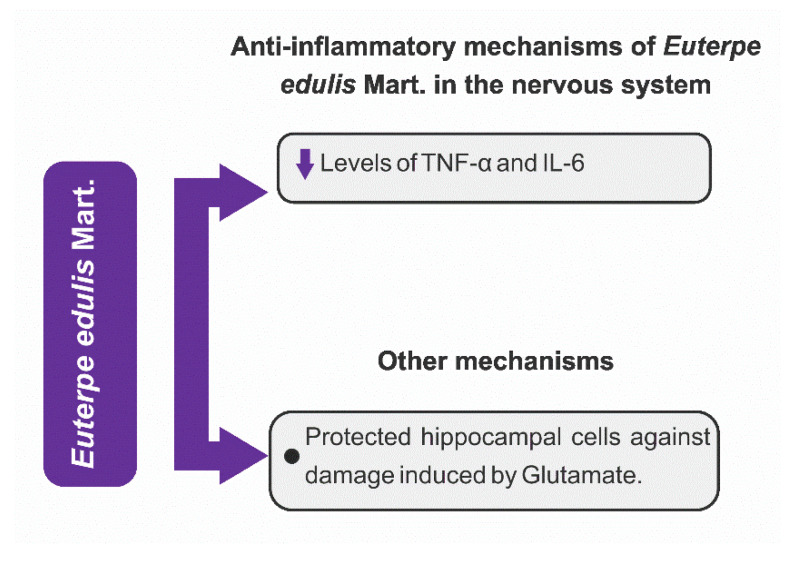
*Euterpe edulis* Mart.—molecular mechanisms of neuroprotection. TNF-α: tumor necrosis factor-α; IL-6: interleukin-6.

Another interesting aspect was the identification of some mechanisms of action through which the fruits and seeds of EO and fruits of EP and EE exerted a neuroprotective effect. From the analysis of the articles included in this review, we observed that the neuroprotection effects of these three *Euterpe* species were mainly due to anti-inflammatory and antioxidant mechanisms, in addition to other mechanisms that are listed in Figure 5, Figure 6 and Figure 7.

**Figure 8:**
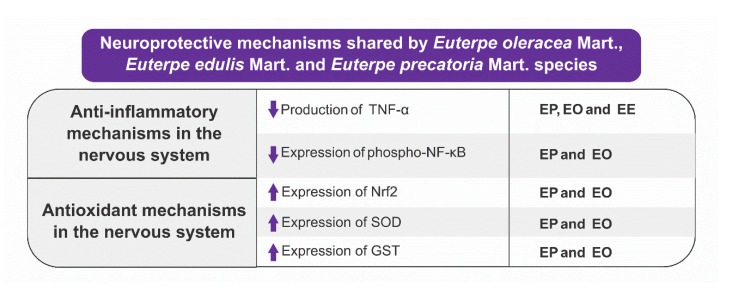
Mechanisms of action shared by *Euterpe oleracea* Mart., *Euterpe precatoria* Mart. And *Euterpe edulis* Mart. species.

A characteristic of the studies selected in this scoping review is that there is no study designed to evaluate the synergistic effect resulting from the combination of AEO, AEP, and JF, even in studies where there was a neuropharmacological evaluation of the two species [51,54,79]. It would be interesting for future neuropharmacological studies to assess the synergistic activity of AEO, AEP, and JF. From this type of evaluation, one would be able to verify whether the neuroprotective effect of the species could be potentiated since AEO, AEP, and JF share some neuroprotective mechanisms (Figure 8).

### 3.3. Oxidative Stress and Neuroinflammation in the Brain

Oxidative stress and neuroinflammation are interconnected pathological events, important in the pathogenesis of several neurodegenerative diseases, as they compromise the integrity of neurons, glial cells, the blood–brain barrier (BBB), and synaptic transmission [34,39,107,108].

Oxidative stress is a consequence of the imbalance between cellular antioxidant defense mechanisms and the generation of pro-oxidant compounds, resulting in the overproduction of free radicals, e.g., reactive oxygen species, reactive nitrogen species, reactive sulfur species (RSS), and electrophiles. Free radicals are atoms or molecules that may contain one or more unpaired electrons and that are characterized by their (1) ability to independently exist, (2) instability, and (3) high reactivity. An important aspect to be highlighted about free radicals is that they can play a dual role depending on their concentration. Physiologically, free radicals are generated from aerobic respiration, and at low or moderate levels, they participate in physiological processes such as the regulation of vascular tone, immune response, and synaptic plasticity [34,35,109,110].

On the other hand, the contribution of free radicals to the development of pathologies is related to their high concentrations since, considering that they are unstable and highly reactive molecules, they can damage living cells through damage to macromolecules (lipids, proteins, RNA, and DNA), leading to lipid peroxidation, denaturation, and loss of function in proteins. In the nervous system, the sum of all these effects can result in synaptic dysfunction and neuronal damage [32,34,36,108,111,112].

Oxidative neuronal damage is one of the main mechanisms involved in the pathogenesis of several neurological disorders, including cerebrovascular and neurodegenerative pathologies, such as Alzheimer’s disease (AD), Parkinson’s disease (PD), stroke, epilepsy, and depression [34,36,112,113]. The significant contribution of oxidative damage to the pathogenesis of these diseases is due to the brain’s particular susceptibility to oxidative stress, which can be explained by factors such as (1) the organ’s chemical composition, (2) the high oxygen consumption, and (3) the brain’s low antioxidant defense when compared to other organs. Regarding its chemical composition, the brain is rich in compounds that participate in the generation of free radicals such as iron ions (an important catalyst for the generation of free radicals, such as the hydroxyl radical through the Fenton reaction) and in polyunsaturated fatty acids of the neuronal cell membrane, which are easily oxidized. Another important aspect to be considered is that the maintenance of cerebral homeostasis demands large amounts of ATP, and this explains why the brain is a voracious consumer of oxygen because, even though it is an organ that represents only 2% to 3% of the body weight, the brain is responsible for consuming 20% of the body’s oxygen and for receiving a volume of blood that corresponds to 15% of the total cardiac output. It is in this scenario that the ambiguity of oxygen is revealed because, even though it is essential for the production of ATP, its high consumption by the brain favors the excessive generation of ROS. Thus, the high levels of free radicals, the limited antioxidant capacity of the brain (low content of antioxidant enzymes, such as catalase content and low cytosolic GSH), the auto-oxidation of neurotransmitters (e.g., dopamine) and the above-mentioned particular conditions make the brain the target organ of oxidative damage [36,38,111,112,114,115,116,117,118,119,120].

Excessive free radical production can result in damage to cellular structures and neuroinflammation, a tissue response characterized by the participation of neurons, glial cells, and BBB dysfunction and by the massive production and release of inflammatory mediators (e.g., cytokines and chemokines) by neurons, glial cells (mainly microglia and astrocytes), tissue damage, and neurodegeneration [39,40,121,122,123,124,125]. This diversity of cells and inflammatory mediators results in neuronal death, astrocytic dysfunction, alteration of neuronal excitability, BBB damage and induction of microglial reactivity, and other morphological and functional impairments to the CNS. The inflammatory response is recognized as a common pathway in the etiopathogenesis of a number of neurological disorders (e.g., epilepsy and multiple sclerosis) and neuropsychiatric disorders, such as depression [125,126,127]. 

Considering that neuroinflammation and oxidative stress are closely related and can be found in many neurological disorders, both events are strategic pharmacological targets for the development of new drugs and/or adjuvant therapies to conventional allopathic treatments [39,43,107,123,125]. According to this idea, our group has recently demonstrated that açaí was able to potentiate the antidepressant activity of imipramine in a model of neuroinflammation [55]. Imipramine is a tricyclic antidepressant whose main mechanism is blocking the monoamine transporters in the nerve endings, resulting in increased concentrations of serotonin and norepinephrine in the synaptic cleft. Considering the possible synergism between açaí and imipramine, it is reasonable to suggest that açaí could have some influence on the monoaminergic system. This hypothesis is reinforced by the modulatory effects that some açaí compounds—such as ellagic acid, ferrulic acid, gallic acid, apigenin, rutin, and resveratrol—can be exerted on the monoaminergic system [128,129,130,131,132,133,134]. Additionally, other mechanisms such as the inhibition of GABA uptake and antiaging effects by increased TERT mRNA expression in the brain (suggesting neuroprotection against long-term age-related disorders) have recently been demonstrated for AEO (Figure 5).

Figure 5 shows that there is great AEO variability mechanism of action; however, the mechanistic studies with AEP and JF showing possible pathways other than neuroinflammation and oxidative stress are extremely scarce (Figure 6 and Figure 7). Certainly, the vast knowledge about the mechanisms through which AEO exerts neuroprotection is linked to the large amount of research about this plant species. If further studies are carried out with EE and EP species, there is the possibility of discovering new mechanisms of action since the fruits of these species are as rich in phenolic compounds as AEO.

### 3.4. Signaling Pathways Targeted by Euterpe Species Associated with Neuroprotection

This article does not intend to describe in detail the pathways or mechanisms involved in neuroprotection by extracts or other products derived from the fruits or seeds of açaí and JF, but rather to identify, map and present a holistic view of the diversity of pathways and mechanisms described in selected articles. This panoramic view of pathways and mechanisms can contribute to the development of studies focused on specific pathways (such as Nrf2) or focused on other signaling pathways that contribute to neuroprotection, but which have not yet been the subject of studies with these species of the *Euterpe* genus.

#### 3.4.1. Anti-Inflammatory Mechanisms

Neuroinflammation is a target of the three *Euterpe* species for neuroprotection. The mechanisms of action and the brain areas protected by AEO, AEP, and JF are shown in Table 1 and Figure 5, Figure 6 and Figure 7. From the analysis of the extracted data, one can observe that (1) AEO can reduce inflammation by several inflammatory pathways, an expected variability due to the greater number of studies with OS; (2) cytokines were the main pharmacological targets of the fruits or seeds of the three studied species. The fruits and seeds of EO were able to reduce phosphorylation and NF-κB activity, the expression of COX-2, and the expression and release of pro-inflammatory cytokines (IL-1β, IL-6, TNF-α). EP and EE fruits exert neuroprotection by decreasing the production of cytokines (TNF-α and IL-6) and expression of NF-κB.

The pro-inflammatory cytokines are promising pharmacological targets since they can favor drug resistance and, at the same time, play a crucial role in the progression of neurological disorders [127,135,136,137,138,139]. In addition to cytokines, it is important that new preclinical and clinical research studies investigate the pharmacological effects of EP and EE (species little explored in relation to their anti-inflammatory effects) on COX-2 and NF-κB.

The knowledge and understanding of the mechanisms of action of these plant species have the potential to create a major impact on the conventional therapy of diseases that affect the nervous system. Some of these diseases (e.g., epilepsy) share high rates of refractoriness to the currently available pharmacological arsenal [140]; in addition, treatments are difficult to access for vulnerable and isolated populations, such as those that exist in the Amazon.

#### 3.4.2. Antioxidant Mechanisms

The antioxidant mechanisms are one of the main pharmacological targets of current neuroprotection research. The antioxidant mechanisms of AEO, AEP, and JF to mitigate oxidative stress are diverse, as they act on complex antioxidant pathways and increase the expression of antioxidant enzymes in the brain.

The analysis of Table 1 shows that AEO and AEP protect the nervous system through several traditional antioxidant mechanisms (increased expression of antioxidant enzymes or free radical scavenger), but in addition to these mechanisms, AEO and AEP induce neuroprotection through the upregulation of transcription nuclear factor erythroid factor 2–related factor 2 (Nrf2), a promising therapeutic target against oxidative stress. This transcription factor regulates the expression of antioxidant genes by binding to the antioxidant response element (ARE) region in DNA, which is considered as a key regulator of antioxidant response [111,141,142,143,144,145,146,147]. In the brain, where it is widely expressed, Nrf2 exerts influence on carbohydrate metabolism, proteostasis, and redox metabolism. The Nrf2 upregulation increases levels of antioxidant enzymes (HO-1, SOD, CAT, among others) that are important for brain protection [141].

Regarding redox metabolism, it is important to emphasize the data of the five studies (see Table 1) that evaluated the effects of the species of the genus *Euterpe* on Nrf2. From the analysis of these manuscripts, we observed that AEO (four studies evaluated the effects of AEO on Nrf2) and AEP increased the expression of Nrf2 in the brain of animals and in cultures of astrocytes, thus inducing antioxidant protection. It is likely that the increase in Nrf2 expression induced neuroprotection through other antioxidant mechanisms observed in these five studies, highlighting the increased expression of GST, SOD, HO-1, and increased antioxidant response element (ARE) activity [31,51,65,72,73]. The increase in Nrf2 expression by AEO and AEP is a very relevant pharmacological effect, as Nrf2 has become a pharmacological target of interest for the treatment of neurodegenerative diseases [141,148,149,150].

AEO and AEP induce neuroprotection through different antioxidant mechanisms (see Table 1, Figure 5 and Figure 6), ranging from increased expression and activity of antioxidant enzymes to reduced levels of nitrites, lipid peroxidation, and protein oxidation. Based on the available literature on the phytochemical composition of the fruits of the EO and EP species, the protection of the nervous system against oxidative stress was already expected since several studies proved that the fruits and even other plant parts (seeds, roots, and leaflets of EO) of these species have a wide diversity of bioactive compounds (e.g., cyanidin 3-glucoside, cyanidin-3-O-rutinoside, resveratrol, apigenin, and luteolin) [18,96,151], but there is an inconsistency when we observed the results, or rather, the absence of neuroprotection by JF based on its already-described antioxidant property [8,10]. It is worth noting that JF is rich in phenolic compounds, has potent antioxidant activity, and is sometimes called a “super food”; hence, it is likely that if there is still no description of neuroprotection associated with antioxidant mechanisms, it is because there is a gap in mechanistic studies with EE species that can be exploited.

## 4. Future Directions

- Corroborating data from preclinical studies, the protective effects of açaí and JF on inflammation and oxidative stress were also observed in clinical studies. Studies in humans with the fruits of the three species of the genus *Euterpe* selected are limited; however, it is worth noting that the clinical studies already published evaluated only the effects of the fruits of EO and EE. So far, there are no studies evaluating the pharmacological effects of EP fruits in humans. Clinical studies with AEO and JF demonstrate that these species are able to reduce inflammatory markers (IL-6, INF-γ) and oxidative stress (8-isoprostane) and increase the activity of antioxidant enzymes (catalase, glutathione peroxidase) in the plasma and serum [9,12,81,152]. Although these effects were observed in clinical conditions unrelated to the CNS, they are important because they demonstrate the protective effects of these fruits in humans and consequently support the need for additional scientific studies, including clinical studies. Furthermore, the multiple mechanisms of action of the *Euterpe* species to exert neuroprotection also support the importance of future research to study the possible application in neurological disorders.
- It would be interesting if future scientific studies addressed, in their experimental designs, in addition to the pharmacodynamic aspects, the evaluation of pharmacokinetic properties (passage through BBB, absorption, distribution, metabolism, and excretion) of products derived (e.g., beverages) from EO, EE, and EP. An important pharmacokinetic property to be investigated in studies with fruits that have a vast phenolic composition is bioavailability since polyphenols have low bioavailability [153,154,155]. In this sense, it would be important to know the pharmacokinetic characteristics of products from EO, EE, and EP so that new discussions and eventual pharmaceutical solutions can be developed to overcome the problems with the bioavailability of polyphenols.
- Considering the data demonstrating that AEO and AEP are able to regulate microglial and astrocytic functions and that these cells perform homeostatic and immune functions in the CNS, it would be relevant to investigate not only the protective functions of AEO, AEP, and JF against brain injury or stimuli to mimic neuroinflammation but also to develop new research that can assess whether AEO, AEP, and JF are able to regulate brain functions under physiological conditions acting on microglia and astrocytes. That is, could EO, EP, or EE contribute to the maintenance of cerebral homeostasis? Could AEO, AEP, or JF modulate neurogenesis? These important questions, as well as others, are still unknown and can certainly contribute to the development of future therapies for brain health.

## 5. Conclusions

- The fruits of EO, EP, or EE species and EO seed extract protect the CNS using mechanisms that reduce/limit the neuroinflammatory process and oxidative stress, and because they are fruits with nutritional and functional appeal and are rich in phenolic compounds and anthocyanins, compounds that exert protective effects through mechanisms common to CNS pathologies, açaí (EO, EP) and juçara (EE) have the potential to impact conventional therapy or even prevent pathologies that affect the CNS.
- EO, EE, and EP species have neuroprotective activity, but this effect is better consolidated in the literature for EO due to the greater amount of *in vitro* and *in vivo* studies.
- The neuroprotection exerted by EO, EE, and EP involves a series of molecular pathways: inhibition of GABA uptake, anti-aging effects, reduction of expression, production, release of inflammatory mediators, potentiation of antioxidant defenses via increased activity and expression of enzymes antioxidants, and reduced ROS production.
- The demand for new knowledge is necessary for the three *Euterpe* species, but based on the available literature evaluated in this article, it is essential that new neuropharmacological studies be directed to EP and EE species, as these two species are rich in phenolic compounds such as flavonoids and phenolic acids.
- In addition to new preclinical studies, there is also a need to carry out clinical studies aimed at evaluating the neuropharmacological activity of these three *Euterpe* species since, to date, there are no clinical studies aimed at evaluating the neuropharmacological activity of EO, EE, and EP. The protection already described in clinical studies with EO and EE (antioxidant and anti-inflammatory effects) is encouraging and may support new clinical studies targeting the CNS.
- It would be relevant if future preclinical and clinical studies were to verify the bioavailability of antioxidant molecules from açaí and juçara pulp or juice in the CNS, which would help determine the effectiveness of these beverages in reducing oxidative stress in the brain and neuroinflammation.
- Despite the low number of studies, one can suggest that açaí and juçara fruit have the potential to impact the therapy of diseases that affect the CNS because they induce neuroprotection through interaction with key pathways (e.g., neuroinflammation and oxidative stress) and alternatives (as autophagy) for the pathogenesis of diseases of the CNS.

## Figures and Tables

**Table 1:** Experimental models and the main results of the studies selected in this scoping review.

*In Vivo* Assays			
Species	Experimental Model/Part of the Plants Used	Outcomes	References
*Euterpe oleracea* Mart.	Model of MeHg intoxication in mice—fruit	- EO ↓ MDA and nitrite levels in the brain; - EO prevented the reduction of the TERT RNA expression in the brain.	[46]
	Depressive-like Behavior induced by LPS in mice—fruit	- Antidepressive effect; - EO ↓ MDA levels in the hippocampus, striatum and prefrontal cortex; - EO ↓ nitrite levels in the hippocampus; - EO ↑ expression of TERT mRNA in the brain of animals with depressive-like behavior; - Prevented neuronal death in the hippocampus.	[55]
	Seizure induced by PTZ in mice—fruit	- Anticonvulsive effect; - EO ↓ MDA levels in the cerebral cortex; - EO ↓ electrical alterations caused by seizures.	[56]
	Pentylenetetrazole (PTZ)-induced seizures in fish—fruit	- Anticonvulsive effect;	[57]
	Seizure induced by PTZ in Rat—Stone	- Anticonvulsive effect via the GABAA receptor;	[58]
	Anxiety induced by periodic maternal separation (PMS) in rats—seed	- Anti-anxiety effect; - EO ↓ MDA and carbonyl levels in the brainstem.	[59]
	Hepatic encephalopathy in rats—fruit	- EO prevented the increase of the cytokines IL-1b, IL-18 and TNF-a in cerebral cortex, hippocampus and cerebellum of rats.	[60]
	Hepatic encephalopathy in rats—fruit	- EO prevented the inhibition of creatine kinase activity (CK) in the cerebral cortex, hippocampus and cerebellum of rats; - EO prevented the enhance of TBARS (cerebral cortex and cerebellum) and carbonyl levels (cerebral cortex, hippocampus and cerebellum); - EO ↑ catalase (CAT) activity in hippocampus and cerebellum; - EO ↑ superoxide dismutase (SOD) activity in the hippocampus.	[61]
	Anorexia-cachexia syndrome induced by Walker-256 tumor in rats—seed	- EO ↓ diameter of the tumor.	[62]
	Evaluation of the effects of EO on learning and memory in rats	- EO improved damaged memory.	[63]
	Infection by *Plasmodium berghei* ANKA strain—fruit	- EO prevented blood–brain barrier (BBB) dysfunction in animals infected with *Plasmodium berghei* ANKA.	[64]
	Experimental model of Parkinson’s disease (PD) MPTP-Induced in mice—Fruit	- EO ↓ degeneration in the brain of animals treated with MPTP; - EO restored dopamine transporter (DAT) levels in the striatum; - EO ↓ expression of Iba-1; - EO ↓ GFAP expression; - EO ↓ release of cytokines TNF-α, IL-1b and IL-6; - EO ↓ MDA levels in the brain; - EO ↑ Nrf2 expression; - EO ↑ HO-1 expression; - EO ↑ SOD expression; - EO ↑ CAT expression; - EO ↑ GPx expression; - EO ↑ GSH expression; - EO ↓ neuronal death.	[31]
	Model of Vascular dementia (VaD) in mice—Fruit	- EO ↓ neuronal death in the hippocampus (CA1 and CA3); - EO ↑ Nrf2 expression in CA1 and CA3; - EO ↑ HO-1 expression in CA1 and CA3; - EO modulated apoptosis and autophagy in the hippocampus of animals submitted to the Model of Vascular dementia (VaD).	[65]
*Euterpe oleracea* Mart. and *Euterpe Precatoria* Mart.	Açaí-enriched diet—fruit	- EP supplementation attenuated NADPH-oxidoreductase-2 (NOX2) expression in rat hippocampus; - EO and EP ↓ expression of phospho-NF-κB expression; - EO and EP ↑ Nrf2 expression in the hippocampus and frontal cortex; - EO and EP ↑ GST expression in the frontal cortex; - EP ↑ GST expression in the hippocampus; - EO and EP ↑ SOD expression in frontal cortex and hippocampus; - EP ↓ levels of mTOR in hippocampus; - EP ↑ beclin1 expression in frontal cortex and hippocampus; - EO ↑ beclin1 expression in the frontal cortex;	[51]
*Euterpe precatoria* Mart.	*Caenorhabditis elegans*—fruit	- EP scavenged the cation radical ABTS (2,2′-azinobis-(3-ethylbenzothiazoline-6-sulfonic acid); - Decreased the number of polyQ aggregates.	[66]
*Euterpe edulis* Mart.	High-Fat Diet—fruit	- EE ↓ levels of Tumor necrosis factor-α (TNF-α) and IL-6 in the hypothalamus.	[67]
***In Vitro* Assays**			
**Species**	**Experimental Model and Part of Plant**	**Outcomes**	**References**
*Euterpe oleracea* Mart.	Tissues treated with hydrogen peroxide (H_2_O_2_)—fruit	- EO ↓ damage in lipids and proteins;	[49]
	Neuronal-like cells SHSY5Y—fruit	- EO ↓ ROS production; - EO reversed rotenone-induced mitochondrial complex I dysfunction; - EO ↓ lipid peroxidation.	[68]
	human neuroblastoma cell line SH-SY5Y—fruit	- Hydroethanolic extracts from EO protected cells against H_2_O_2_.	[69]
	Primary hippocampal neurons and HT22 mouse hippocampal cells—fruit	- EO and EP ↓ accumulation of autophagic vacuoles in HT22 mouse hippocampal neurons; - EO and EP ↓ levels of pospho-mTOR in the HT22 mouse hippocampal neurons.	[54]
	Rat phaeochromocytoma cells (PC12 cell)—fruit	- EO inhibited the loss of cell viability.	[70]
	C-6 rat brain carcinoma cells—fruit	- EO ↓ proliferation of C-6 cells; - EO induced DNA fragmentation.	[71]
	Primary Cultures of Rat Astrocytes—fruit	- EO protected cells against lipid peroxidation;	[72]
	Immortalized rat astrocytes (DI TNC1)—fruit	- EO inhibited the NF-κB activity LPS-induced; - EO ↑ Antioxidant Response Element (ARE) activity; - EO ↑ Expression of Nrf2 and HO-1.	[73]
	Primary cultures of cortical neurons and astrocytes—fruit	- EO ↓ [^3^H]TBOB binding on the GABAA receptor in cortical neurons; - EO ↑ [^3^H]flunitrazepam binding on the GABAA receptor in neuronal cultures; - EO inhibited GABA uptake in both cortical neurons and astrocytes;	[74]
	BV-2 microglia cell line—fruit	- EO ↓ cell proliferation; - EO ↓ ROS production; - EO ↓ expression of pro-inflammatory cytokines (IL-1β, IL-6, TNF-α); - EO ↓ expression of caspases (Casp1, Casp3 and Casp8).	[75]
	Microglia EOC 13.31 cell line—fruit	- EO ↓ expression of IL-1β;	[76]
	BV-2 microglia cell line—fruit	- EO ↓ iNOS expression; - EO ↓ release of the cytokine TNF-α; - EO attenuated p38-MAPK phosphorylation; - EO ↓ the phosphorylation of NF-κB; - EO ↓ COX-2 expression.	[77]
*Euterpe edulis* Mart.	Mouse hippocampal HT22 cells—fruit	- EE protected hippocampal cells against glutamate-induced oxytosis.	[78]
***In Vitro* and *In Vivo* Assays**			
**Species**	**Experimental Model**	**Outcomes**	**References**
*Euterpe oleracea* Mart. and *Euterpe precatoria* Mart.	Dietary supplementation with EO and EP—*in vivo*—fruit BV-2 cells were treated with blood serum from both EO- and EP-fed rats—*in vitro*—fruit	- Microglial cells treated with blood serum from EO-fed animals produced less NO; - iNOS expression was attenuated in microglial cells treated with blood serum from animals fed EO and EP; - The production of TNF-α was attenuated in microglial cells treated with blood serum from animals fed with EO and EP.	[79]

**Abbreviations**: EO: *Euterpe oleracea* Mart.; EE: *Euterpe edulis* Mart.; and EP: *Euterpe precatoria* Mart.

## Data Availability

Not applicable.

## References

[1] Melzer T.M., Manosso L.M., Yau S.Y., Gil-Mohapel J., Brocardo P.S. (2021). In Pursuit of Healthy Aging: Effects of Nutrition on Brain Function. Int. J. Mol. Sci..

[2] Henriques J.F., Serra D., Dinis T.C.P., Almeida L.M. (2020). The Anti-Neuroinflammatory Role of Anthocyanins and Their Metabolites for the Prevention and Treatment of Brain Disorders. Int. J. Mol. Sci..

[3] Winter A.N., Bickford P.C. (2019). Anthocyanins and Their Metabolites as Therapeutic Agents for Neurodegenerative Disease. Antioxidants.

[4] Vauzour D. (2012). Dietary polyphenols as modulators of brain functions: Biological actions and molecular mechanisms underpinning their beneficial effects. Oxid. Med. Cell. Longev..

[5] Chang S.K., Alasalvar C., Shahidi F. (2019). Superfruits: Phytochemicals, antioxidant efficacies, and health effects—A comprehensive review. Crit. Rev. Food Sci. Nutr..

[6] Neri-Numa I.A., Soriano Sancho R.A., Pereira A.P.A., Pastore G.M. (2018). Small Brazilian wild fruits: Nutrients, bioactive compounds, health-promotion properties and commercial interest. Food Res. Int..

[7] Pacheco-Palencia L.A., Duncan C.E., Talcott S.T. (2009). Phytochemical composition and thermal stability of two commercial açai species, *Euterpe oleracea* and Euterpe precatoria. Food Chem..

[8] Bicudo M.O., Ribani R.H., Beta T. (2014). Anthocyanins, phenolic acids and antioxidant properties of Jucara fruits (*Euterpe edulis* M.) along the on-tree ripening process. Plant Foods Hum. Nutr..

[9] Aranha L.N., Silva M.G., Uehara S.K., Luiz R.R., Nogueira Neto J.F., Rosa G., Moraes de Oliveira G.M. (2020). Effects of a hypoenergetic diet associated with acai (*Euterpe oleracea* Mart.) pulp consumption on antioxidant status, oxidative stress and inflammatory biomarkers in overweight, dyslipidemic individuals. Clin. Nutr..

[10] Cardoso A.L., de Liz S., Rieger D.K., Farah A.C.A., Kunradi Vieira F.G., Altenburg de Assis M.A., Di Pietro P.F. (2018). An Update on the Biological Activities of *Euterpe edulis* (Jucara). Planta Med..

[11] Jensen G.S., Wu X., Patterson K.M., Barnes J., Carter S.G., Scherwitz L., Beaman R., Endres J.R., Schauss A.G. (2008). *In vitro* and *in vivo* antioxidant and anti-inflammatory capacities of an antioxidant-rich fruit and berry juice blend. Results of a pilot and randomized, double-blinded, placebo-controlled, crossover study. J. Agric. Food Chem..

[12] Kim H., Simbo S.Y., Fang C., McAlister L., Roque A., Banerjee N., Talcott S.T., Zhao H., Kreider R.B., Mertens-Talcott S.U. (2018). Acai (*Euterpe oleracea* Mart.) beverage consumption improves biomarkers for inflammation but not glucose- or lipid-metabolism in individuals with metabolic syndrome in a randomized, double-blinded, placebo-controlled clinical trial. Food Funct..

[13] Vianna S.A. Euterpe in Flora do Brasil. https://floradobrasil.jbrj.gov.br/FB15713.

[14] Vianna S.A. Euterpe in Flora do Brasil. https://floradobrasil.jbrj.gov.br/FB22139.

[15] Vianna S.A. Euterpe in Flora do Brasil. https://floradobrasil.jbrj.gov.br/FB15712.

[16] Brasil Catálago de Produtos da Sociobiodiversidade do Brasil. https://www.gov.br/icmbio/pt-br/centrais-de-conteudo/publicacoes/publicacoes-diversas/catalago_de_produtos_da_sociobiodiversidade_do_brasil.pdf/view.

[17] Brasil Alimentos Regionais Brasileiros. https://bvsms.saude.gov.br/bvs/publicacoes/alimentos_regionais_brasileiros_2ed.pdf.

[18] Yamaguchi K.K., Pereira L.F., Lamarao C.V., Lima E.S., da Veiga-Junior V.F. (2015). Amazon acai: Chemistry and biological activities: A review. Food Chem..

[19] Oliveira M.D.S.P.D., Schwartz G., Rodrigues S., Silva E., de Brito E. (2018). Açaí—*Euterpe oleracea*. Exotic Fruits Reference Guide.

[20] Schauss A.G., Watson R.R., Preedy V.R. (2015). The Effect of Acai (*Euterpe* spp.) Fruit Pulp on Brain Health and Performance. Bioactive Nutraceuticals and Dietary Supplements in Neurological and Brain Disease.

[21] Boeira L.S., Freitas P.H.B., Uchôa N.R., Bezerra J.A., Cád S.V., Junior S.D., Albuquerque P.M., Mar J.M., Ramos A.S., Machado M.B. (2020). Chemical and sensorial characterization of a novel alcoholic beverage produced with native acai (Euterpe precatoria) from different regions of the Amazonas state. Food Sci. Technol..

[22] Borges G.D.S.C., Gonzaga L.V., Jardini F.A., Filho J.M., Heller M., Micke G., Costa A.C.O., Fett R. (2013). Protective effect of *Euterpe edulis* M. on Vero cell culture and antioxidant evaluation based on phenolic composition using HPLC−ESI-MS/MS. Food Res. Int..

[23] Schulz M., da Silva Campelo Borges G., Gonzaga L.V., Oliveira Costa A.C., Fett R. (2016). Jucara fruit (*Euterpe edulis* Mart.): Sustainable exploitation of a source of bioactive compounds. Food Res. Int..

[24] Trevisan A.C.D., Fantini A.C., Schmitt-Filho A.L., Farley J. (2015). Market for Amazonian Açaí (*Euterpe oleraceae*) Stimulates Pulp Production from Atlantic Forest Juçara Berries (*Euterpe edulis*). Agroecol. Sustain. Food Syst..

[25] Schulz M., Borges G.D.S.C., Gonzaga L.V., Seraglio S.K.T., Olivo I.S., Azevedo M.S., Nehring P., de Gois J.S., de Almeida T.S., Vitali L. (2015). Chemical composition, bioactive compounds and antioxidant capacity of juçara fruit (*Euterpe edulis* Martius) during ripening. Food Res. Int..

[26] Schauss A.G., Alasalvar C., Shahidi F. (2013). Açai fruits: Potent antioxidant and anti-inflammatory superfruits with potential health benefits. Dried Fruits: Phytochemicals and Health Effects.

[27] Vannuchi N., Jamar G., Pisani L., Braga A.R.C., de Rosso V.V. (2021). Chemical composition, bioactive compounds extraction, and observed biological activities from jussara (*Euterpe edulis*): The exotic and endangered Brazilian superfruit. Compr. Rev. Food Sci. Food Saf..

[28] CONAB—Companhia Nacional De Abastecimento Boletim da Sociobiodiversidade, Brasília, DF, v. 5, n. 5, Outubro 2021., 23. https://www.conab.gov.br/info-agro/analises-do-mercado-agropecuario-e-extrativista/boletim-da-sociobiodiversidade/boletim-sociobio.

[29] Schauss A.G., Wu X., Prior R.L., Ou B., Patel D., Huang D., Kababick J.P. (2006). Phytochemical and nutrient composition of the freeze-dried amazonian palm berry, *Euterpe oleraceae* mart. (acai). J. Agric. Food Chem..

[30] Gordon A., Cruz A.P., Cabral L.M., de Freitas S.C., Taxi C.M., Donangelo C.M., de Andrade Mattietto R., Friedrich M., da Matta V.M., Marx F. (2012). Chemical characterization and evaluation of antioxidant properties of acai fruits (*Euterpe oleraceae* Mart.) during ripening. Food Chem..

[31] D’Amico R., Impellizzeri D., Genovese T., Fusco R., Peritore A.F., Crupi R., Interdonato L., Franco G., Marino Y., Arangia A. (2022). Acai Berry Mitigates Parkinson’s Disease Progression Showing Dopaminergic Neuroprotection via Nrf2-HO1 Pathways. Mol. Neurobiol..

[32] Cardenas-Rodriguez N., Huerta-Gertrudis B., Rivera-Espinosa L., Montesinos-Correa H., Bandala C., Carmona-Aparicio L., Coballase-Urrutia E. (2013). Role of oxidative stress in refractory epilepsy: Evidence in patients and experimental models. Int. J. Mol. Sci..

[33] Popa-Wagner A., Mitran S., Sivanesan S., Chang E., Buga A.M. (2013). ROS and brain diseases: The good, the bad, and the ugly. Oxid. Med. Cell. Longev..

[34] Singh E., Devasahayam G. (2020). Neurodegeneration by oxidative stress: A review on prospective use of small molecules for neuroprotection. Mol. Biol. Rep..

[35] Phaniendra A., Jestadi D.B., Periyasamy L. (2015). Free radicals: Properties, sources, targets, and their implication in various diseases. Indian J. Clin. Biochem..

[36] Lee K.H., Cha M., Lee B.H. (2020). Neuroprotective Effect of Antioxidants in the Brain. Int. J. Mol. Sci..

[37] Rekatsina M., Paladini A., Piroli A., Zis P., Pergolizzi J.V., Varrassi G. (2020). Pathophysiology and Therapeutic Perspectives of Oxidative Stress and Neurodegenerative Diseases: A Narrative Review. Adv. Ther..

[38] Cobley J.N., Fiorello M.L., Bailey D.M. (2018). 13 reasons why the brain is susceptible to oxidative stress. Redox Biol..

[39] Lyman M., Lloyd D.G., Ji X., Vizcaychipi M.P., Ma D. (2014). Neuroinflammation: The role and consequences. Neurosci. Res..

[40] Kumar V. (2019). Toll-like receptors in the pathogenesis of neuroinflammation. J. Neuroimmunol..

[41] Jin R., Liu L., Zhang S., Nanda A., Li G. (2013). Role of inflammation and its mediators in acute ischemic stroke. J. Cardiovasc. Transl. Res..

[42] Solleiro-Villavicencio H., Rivas-Arancibia S. (2018). Effect of Chronic Oxidative Stress on Neuroinflammatory Response Mediated by CD4(+)T Cells in Neurodegenerative Diseases. Front. Cell Neurosci..

[43] Biswas S.K. (2016). Does the Interdependence between Oxidative Stress and Inflammation Explain the Antioxidant Paradox?. Oxid. Med. Cell. Longev..

[44] Terrone G., Balosso S., Pauletti A., Ravizza T., Vezzani A. (2020). Inflammation and reactive oxygen species as disease modifiers in epilepsy. Neuropharmacology.

[45] Pajares M., Rojo A.I., Manda G., Boscá L., Cuadrado A. (2020). Inflammation in Parkinson’s Disease: Mechanisms and Therapeutic Implications. Cells.

[46] Crespo-Lopez M.E., Soares E.S., Macchi B.M., Santos-Sacramento L., Takeda P.Y., Lopes-Araujo A., Paraense R.S.O., Souza-Monteiro J.R., Augusto-Oliveira M., Luz D.A. (2019). Towards Therapeutic Alternatives for Mercury Neurotoxicity in the Amazon: Unraveling the Pre-Clinical Effects of the Superfruit Acai (*Euterpe oleracea*, Mart.) as Juice for Human Consumption. Nutrients.

[47] Farina M., Rocha J.B., Aschner M. (2011). Mechanisms of methylmercury-induced neurotoxicity: Evidence from experimental studies. Life Sci..

[48] Tricco A.C., Lillie E., Zarin W., O’Brien K.K., Colquhoun H., Levac D., Moher D., Peters M.D.J., Horsley T., Weeks L. (2018). PRISMA Extension for Scoping Reviews (PRISMA-ScR): Checklist and Explanation. Ann. Intern. Med..

[49] Spada P.D., Dani C., Bortolini G.V., Funchal C., Henriques J.A., Salvador M. (2009). Frozen fruit pulp of *Euterpe oleraceae* Mart. (Acai) prevents hydrogen peroxide-induced damage in the cerebral cortex, cerebellum, and hippocampus of rats. J. Med. Food.

[50] CONAB (2019). Açaí-Análise Mensal-Março. https://www.conab.gov.br/info-agro/analises-do-mercado-agropecuario-e-extrativista/analises-do-mercado/historico-mensal-de-acai.

[51] Poulose S.M., Bielinski D.F., Carey A., Schauss A.G., Shukitt-Hale B. (2017). Modulation of oxidative stress, inflammation, autophagy and expression of Nrf2 in hippocampus and frontal cortex of rats fed with acai-enriched diets. Nutr. Neurosci..

[52] Heinrich M., Dhanji T., Casselman I. (2011). Açai (*Euterpe oleracea* Mart.)—A phytochemical and pharmacological assessment of the species’ health claims. Phytochem. Lett..

[53] Kang J., Thakali K.M., Xie C., Kondo M., Tong Y., Ou B., Jensen G., Medina M.B., Schauss A.G., Wu X. (2012). Bioactivities of açaí (Euterpe precatoria Mart.) fruit pulp, superior antioxidant and anti-inflammatory properties to *Euterpe oleracea* Mart. Food Chem..

[54] Poulose S.M., Fisher D.R., Bielinski D.F., Gomes S.M., Rimando A.M., Schauss A.G., Shukitt-Hale B. (2014). Restoration of stressor-induced calcium dysregulation and autophagy inhibition by polyphenol-rich acai (*Euterpe* spp.) fruit pulp extracts in rodent brain cells *in vitro*. Nutrition.

[55] Souza-Monteiro J.R., Arrifano G.P.F., Queiroz A., Mello B.S.F., Custodio C.S., Macedo D.S., Hamoy M., Paraense R.S.O., Bittencourt L.O., Lima R.R. (2019). Antidepressant and Antiaging Effects of Acai (*Euterpe oleracea* Mart.) in Mice. Oxid. Med. Cell. Longev..

[56] Souza-Monteiro J.R., Hamoy M., Santana-Coelho D., Arrifano G.P., Paraense R.S., Costa-Malaquias A., Mendonca J.R., da Silva R.F., Monteiro W.S., Rogez H. (2015). Anticonvulsant properties of *Euterpe oleracea* in mice. Neurochem. Int..

[57] da Silva T.V.N., Torres M.F., Sampaio L.A., Hamoy M., Monserrat J.M., Barbas L.A.L. (2021). Dietary *Euterpe oleracea* Mart. attenuates seizures and damage to lipids in the brain of Colossoma macropomum. Fish Physiol. Biochem..

[58] Muto N.A., Hamoy M., da Silva Ferreira C.B., Hamoy A.O., Lucas D.C.R., de Mello V.J., Rogez H. (2022). Extract of *Euterpe oleracea* Martius Stone Presents Anticonvulsive Activity via the GABAA Receptor. Front. Cell Neurosci..

[59] de Bem G.F., Okinga A., Ognibene D.T., da Costa C.A., Santos I.B., Soares R.A., Silva D.L.B., da Rocha A.P.M., Isnardo Fernandes J., Fraga M.C. (2020). Anxiolytic and antioxidant effects of *Euterpe oleracea* Mart. (acai) seed extract in adult rat offspring submitted to periodic maternal separation. Appl. Physiol. Nutr. Metab..

[60] de Souza Machado F., Marinho J.P., Abujamra A.L., Dani C., Quincozes-Santos A., Funchal C. (2015). Carbon Tetrachloride Increases the Pro-inflammatory Cytokines Levels in Different Brain Areas of Wistar Rats: The Protective Effect of Acai Frozen Pulp. Neurochem. Res..

[61] de Souza Machado F., Kuo J., Wohlenberg M.F., da Rocha Frusciante M., Freitas M., Oliveira A.S., Andrade R.B., Wannmacher C.M., Dani C., Funchal C. (2016). Subchronic treatment with acai frozen pulp prevents the brain oxidative damage in rats with acute liver failure. Metab. Brain Dis..

[62] Nascimento V.H., Lima C.D., Paixao J.T., Freitas J.J., Kietzer K.S. (2016). Antioxidant effects of acai seed ( *Euterpe oleracea*) in anorexia-cachexia syndrome induced by Walker-256 tumor. Acta Cir. Bras..

[63] Yildirim C., Aydin S., Donertas B., Oner S., Kilic F.S. (2020). Effects of *Euterpe oleracea* to Enhance Learning and Memory in a Conditioned Nicotinic and Muscarinic Receptor Response Paradigm by Modulation of Cholinergic Mechanisms in Rats. J. Med. Food.

[64] Oliveira K., Torres M.L.M., Kauffmann N., de Azevedo Ataide B.J., de Souza Franco Mendes N., Dos Anjos L.M., Dos Santos Borges R., Bahia C.P., Leao L.K.R., da Conceicao Fonseca Passos A. (2022). *Euterpe oleracea* fruit (Acai)-enriched diet suppresses the development of experimental cerebral malaria induced by Plasmodium berghei (ANKA) infection. BMC Complement. Med. Ther..

[65] Impellizzeri D., D’Amico R., Fusco R., Genovese T., Peritore A.F., Gugliandolo E., Crupi R., Interdonato L., Di Paola D., Di Paola R. (2022). Acai Berry Mitigates Vascular Dementia-Induced Neuropathological Alterations Modulating Nrf-2/Beclin1 Pathways. Cells.

[66] Peixoto H., Roxo M., Krstin S., Wang X., Wink M. (2016). Anthocyanin-rich extract of Acai (Euterpe precatoria Mart.) mediates neuroprotective activities in Caenorhabditis elegans. J. Funct. Foods.

[67] Santamarina A.B., Jamar G., Mennitti L.V., de Rosso V.V., Cesar H.C., Oyama L.M., Pisani L.P. (2018). The Use of Jucara (*Euterpe edulis* Mart.) Supplementation for Suppression of NF-kappaB Pathway in the Hypothalamus after High-Fat Diet in Wistar Rats. Molecules.

[68] Machado A.K., Andreazza A.C., da Silva T.M., Boligon A.A., do Nascimento V., Scola G., Duong A., Cadona F.C., Ribeiro E.E., da Cruz I.B. (2016). Neuroprotective Effects of Acai (*Euterpe oleracea* Mart.) against Rotenone In Vitro Exposure. Oxid. Med. Cell. Longev..

[69] Torma P.D., Brasil A.V., Carvalho A.V., Jablonski A., Rabelo T.K., Moreira J.C., Gelain D.P., Flores S.H., Augusti P.R., Rios A.O. (2017). Hydroethanolic extracts from different genotypes of acai (*Euterpe oleracea*) presented antioxidant potential and protected human neuron-like cells (SH-SY5Y). Food Chem..

[70] Wong D.Y., Musgrave I.F., Harvey B.S., Smid S.D. (2013). Acai (*Euterpe oleraceae* Mart.) berry extract exerts neuroprotective effects against beta-amyloid exposure in vitro. Neurosci. Lett..

[71] Hogan S., Chung H., Zhang L., Li J., Lee Y., Dai Y., Zhou K. (2010). Antiproliferative and antioxidant properties of anthocyanin-rich extract from açai. Food Chem..

[72] da Silva Santos V., Bisen-Hersh E., Yu Y., Cabral I.S., Nardini V., Culbreth M., Teixeira da Rocha J.B., Barbosa F., Aschner M. (2014). Anthocyanin-rich acai (*Euterpe oleracea* Mart.) extract attenuates manganese-induced oxidative stress in rat primary astrocyte cultures. J. Toxicol. Environ. Health A.

[73] Ajit D., Simonyi A., Li R., Chen Z., Hannink M., Fritsche K.L., Mossine V.V., Smith R.E., Dobbs T.K., Luo R. (2016). Phytochemicals and botanical extracts regulate NF-kappaB and Nrf2/ARE reporter activities in DI TNC1 astrocytes. Neurochem. Int..

[74] Arrifano G.P.F., Lichtenstein M.P., Souza-Monteiro J.R., Farina M., Rogez H., Carvalho J.C.T., Sunol C., Crespo-Lopez M.E. (2018). Clarified Acai (*Euterpe oleracea*) Juice as an Anticonvulsant Agent: In Vitro Mechanistic Study of GABAergic Targets. Oxid. Med. Cell. Longev..

[75] de Souza D.V., Pappis L., Bandeira T.T., Sangoi G.G., Fontana T., Rissi V.B., Sagrillo M.R., Duarte M.M., Duarte T., Bodenstein D.F. (2022). Acai (*Euterpe oleracea* Mart.) presents anti-neuroinflammatory capacity in LPS-activated microglia cells. Nutr. Neurosci..

[76] Cadona F.C., de Souza D.V., Fontana T., Bodenstein D.F., Ramos A.P., Sagrillo M.R., Salvador M., Mota K., Davidson C.B., Ribeiro E.E. (2021). Acai (*Euterpe oleracea* Mart.) as a Potential Anti-neuroinflammatory Agent: NLRP3 Priming and Activating Signal Pathway Modulation. Mol. Neurobiol..

[77] Poulose S.M., Fisher D.R., Larson J., Bielinski D.F., Rimando A.M., Carey A.N., Schauss A.G., Shukitt-Hale B. (2012). Anthocyanin-rich acai (*Euterpe oleracea* Mart.) fruit pulp fractions attenuate inflammatory stress signaling in mouse brain BV-2 microglial cells. J. Agric. Food Chem..

[78] Schulz M., Gonzaga L.V., de Souza V., Farina M., Vitali L., Micke G.A., Costa A.C.O., Fett R. (2019). Neuroprotective effect of jucara (*Euterpe edulis* Martius) fruits extracts against glutamate-induced oxytosis in HT22 hippocampal cells. Food Res. Int..

[79] Carey A.N., Miller M.G., Fisher D.R., Bielinski D.F., Gilman C.K., Poulose S.M., Shukitt-Hale B. (2017). Dietary supplementation with the polyphenol-rich acai pulps (*Euterpe oleracea* Mart. and Euterpe precatoria Mart.) improves cognition in aged rats and attenuates inflammatory signaling in BV-2 microglial cells. Nutr. Neurosci..

[80] Goh J.-Y., Weaver R.J., Dixon L., Platt N.J., Roberts R.A. (2015). Development and use of in vitro alternatives to animal testing by the pharmaceutical industry 1980–2013. Toxicol. Res..

[81] de Liz S., Cardoso A.L., Copetti C.L.K., Hinnig P.F., Vieira F.G.K., da Silva E.L., Schulz M., Fett R., Micke G.A., Di Pietro P.F. (2020). Acai (*Euterpe oleracea* Mart.) and jucara (*Euterpe edulis* Mart.) juices improved HDL-c levels and antioxidant defense of healthy adults in a 4-week randomized cross-over study. Clin. Nutr..

[82] Pala D., Barbosa P.O., Silva C.T., de Souza M.O., Freitas F.R., Volp A.C.P., Maranhao R.C., Freitas R.N. (2018). Acai (*Euterpe oleracea* Mart.) dietary intake affects plasma lipids, apolipoproteins, cholesteryl ester transfer to high-density lipoprotein and redox metabolism: A prospective study in women. Clin. Nutr..

[83] Vigneron M., Deparis X., Deharo E., Bourdy G. (2005). Antimalarial remedies in French Guiana: A knowledge attitudes and practices study. J. Ethnopharmacol..

[84] Kffuri C.W., Lopes M.A., Ming L.C., Odonne G., Kinupp V.F. (2016). Antimalarial plants used by indigenous people of the Upper Rio Negro in Amazonas, Brazil. J. Ethnopharmacol..

[85] Gois M.A.F., Lucas F.C.A., Costa J.C.M., Moura P.H.B.D., Lobato G.D.J.M. (2016). Etnobotânica de espécies vegetais medicinais no tratamento de transtornos do sistema gastrointestinal. Rev. Bras. Plant. Med..

[86] Martins A.G., Rosário D.L.d., Barros M.N.d., Jardim M.A.G. (2005). Etnobotanical research of medicinal, alimentary and toxic plants in Combu Island, County of Belém, Pará, Brazil. Rev. Bras. Farm..

[87] Pereira M.D.G.D.S., Coelho-Ferreira M. (2017). Uso e diversidade de plantas medicinais em uma comunidade quilombola na Amazônia Oriental, Abaetetuba, Pará. Biota Amaz..

[88] Jardim M.A.G., Medeiros T.D.S. (2006). Plantas oleaginosas do Estado do Pará: Composição florística e usos medicinais. Rev. Bras. Farmácia.

[89] Carneiro F.M., Silva M.J.P.d., Borges L.L., Albernaz L.C., Costa J.D.P. (2014). Trends of studies for medicinal plants in Brazil. Rev. Sapiência Soc. Saberes E Práticas Educ..

[90] da Silva Cristino Cordeiro V., de Bem G.F., da Costa C.A., Santos I.B., de Carvalho L., Ognibene D.T., da Rocha A.P.M., de Carvalho J.J., de Moura R.S., Resende A.C. (2018). *Euterpe oleracea* Mart. seed extract protects against renal injury in diabetic and spontaneously hypertensive rats: Role of inflammation and oxidative stress. Eur. J. Nutr..

[91] de Andrade Soares R., de Oliveira B.C., de Bem G.F., de Menezes M.P., Romao M.H., Santos I.B., da Costa C.A., de Carvalho L., Nascimento A.L.R., de Carvalho J.J. (2020). Acai (*Euterpe oleracea* Mart.) seed extract improves aerobic exercise performance in rats. Food Res. Int..

[92] Martins G.R., Guedes D., Marques de Paula U.L., de Oliveira M., Lutterbach M.T.S., Reznik L.Y., Servulo E.F.C., Alviano C.S., Ribeiro da Silva A.J., Alviano D.S. (2021). Acai (*Euterpe oleracea* Mart.) Seed Extracts from Different Varieties: A Source of Proanthocyanidins and Eco-Friendly Corrosion Inhibition Activity. Molecules.

[93] Rauf A., Imran M., Abu-Izneid T., Iahtisham Ul H., Patel S., Pan X., Naz S., Sanches Silva A., Saeed F., Rasul Suleria H.A. (2019). Proanthocyanidins: A comprehensive review. Biomed. Pharmacother..

[94] Sova M., Saso L. (2020). Natural Sources, Pharmacokinetics, Biological Activities and Health Benefits of Hydroxycinnamic Acids and Their Metabolites. Nutrients.

[95] Wang X., Cao Y., Chen S., Lin J., Bian J., Huang D. (2021). Anti-Inflammation Activity of Flavones and Their Structure-Activity Relationship. J. Agric. Food Chem..

[96] Matheus M.E., de Oliveira Fernandes S.B., Silveira C.S., Rodrigues V.P., de Sousa Menezes F., Fernandes P.D. (2006). Inhibitory effects of *Euterpe oleracea* Mart. on nitric oxide production and iNOS expression. J. Ethnopharmacol..

[97] Jha M.K., Jo M., Kim J.H., Suk K. (2019). Microglia-Astrocyte Crosstalk: An Intimate Molecular Conversation. Neuroscientist.

[98] Augusto-Oliveira M., Arrifano G.P., Lopes-Araujo A., Santos-Sacramento L., Takeda P.Y., Anthony D.C., Malva J.O., Crespo-Lopez M.E. (2019). What Do Microglia Really Do in Healthy Adult Brain?. Cells.

[99] Colonna M., Butovsky O. (2017). Microglia Function in the Central Nervous System During Health and Neurodegeneration. Annu. Rev. Immunol..

[100] Augusto-Oliveira M., Arrifano G.P., Takeda P.Y., Lopes-Araujo A., Santos-Sacramento L., Anthony D.C., Verkhratsky A., Crespo-Lopez M.E. (2020). Astroglia-specific contributions to the regulation of synapses, cognition and behaviour. Neurosci. Biobehav. Rev..

[101] Crespo-Lopez M.E., Herculano A.M., Corvelo T.C., Do Nascimento J.L. (2005). Mercury and neurotoxicity. Rev. Neurol..

[102] Berzas Nevado J.J., Rodriguez Martin-Doimeadios R.C., Guzman Bernardo F.J., Jimenez Moreno M., Herculano A.M., do Nascimento J.L., Crespo-Lopez M.E. (2010). Mercury in the Tapajos River basin, Brazilian Amazon: A review. Environ. Int..

[103] Arrifano G.P.F., Martin-Doimeadios R.C.R., Jimenez-Moreno M., Ramirez-Mateos V., da Silva N.F.S., Souza-Monteiro J.R., Augusto-Oliveira M., Paraense R.S.O., Macchi B.M., do Nascimento J.L.M. (2018). Large-scale projects in the amazon and human exposure to mercury: The case-study of the Tucurui Dam. Ecotoxicol. Environ. Saf..

[104] Rodriguez Martin-Doimeadios R.C., Berzas Nevado J.J., Guzman Bernardo F.J., Jimenez Moreno M., Arrifano G.P., Herculano A.M., do Nascimento J.L., Crespo-Lopez M.E. (2014). Comparative study of mercury speciation in commercial fishes of the Brazilian Amazon. Environ. Sci. Pollut. Res. Int..

[105] Kang J., Li Z., Wu T., Jensen G.S., Schauss A.G., Wu X. (2010). Anti-oxidant capacities of flavonoid compounds isolated from acai pulp (*Euterpe oleracea* Mart.). Food Chem..

[106] de Almeida Magalhaes T.S.S., de Oliveira Macedo P.C., Converti A., Neves de Lima A.A. (2020). The Use of *Euterpe oleracea* Mart. As a New Perspective for Disease Treatment and Prevention. Biomolecules.

[107] Pape K., Tamouza R., Leboyer M., Zipp F. (2019). Immunoneuropsychiatry—Novel perspectives on brain disorders. Nat. Rev. Neurol..

[108] Kamat P.K., Kalani A., Rai S., Swarnkar S., Tota S., Nath C., Tyagi N. (2016). Mechanism of Oxidative Stress and Synapse Dysfunction in the Pathogenesis of Alzheimer’s Disease: Understanding the Therapeutics Strategies. Mol. Neurobiol..

[109] Poprac P., Jomova K., Simunkova M., Kollar V., Rhodes C.J., Valko M. (2017). Targeting Free Radicals in Oxidative Stress-Related Human Diseases. Trends Pharmacol. Sci..

[110] Valko M., Leibfritz D., Moncol J., Cronin M.T., Mazur M., Telser J. (2007). Free radicals and antioxidants in normal physiological functions and human disease. Int. J. Biochem. Cell Biol..

[111] Hannan M.A., Dash R., Sohag A.A.M., Haque M.N., Moon I.S. (2020). Neuroprotection Against Oxidative Stress: Phytochemicals Targeting TrkB Signaling and the Nrf2-ARE Antioxidant System. Front. Mol. Neurosci..

[112] Geronzi U., Lotti F., Grosso S. (2018). Oxidative stress in epilepsy. Expert Rev. Neurother..

[113] Teleanu D.M., Niculescu A.G., Lungu I.l., Radu C.I., Vladacenco O., Roza E., Costachescu B., Grumezescu A.M., Teleanu R.I. (2022). An Overview of Oxidative Stress, Neuroinflammation, and Neurodegenerative Diseases. Int. J. Mol. Sci..

[114] Morella I.M., Brambilla R., More L. (2022). Emerging roles of brain metabolism in cognitive impairment and neuropsychiatric disorders. Neurosci. Biobehav. Rev..

[115] Faria-Pereira A., Morais V.A. (2022). Synapses: The Brain’s Energy-Demanding Sites. Int. J. Mol. Sci..

[116] Du F., Zhu X.H., Zhang Y., Friedman M., Zhang N., Ugurbil K., Chen W. (2008). Tightly coupled brain activity and cerebral ATP metabolic rate. Proc. Natl. Acad. Sci. USA.

[117] Takahashi S. (2021). Neuroprotective Function of High Glycolytic Activity in Astrocytes: Common Roles in Stroke and Neurodegenerative Diseases. Int. J. Mol. Sci..

[118] Takahashi S. (2022). Metabolic Contribution and Cerebral Blood Flow Regulation by Astrocytes in the Neurovascular Unit. Cells.

[119] Claassen J.A.H.R., Dick H.J., Thijssen R.B.P., Faraci F.M. (2021). Regulation of cerebral blood flow in humans: Physiology and clinical implications of autoregulation. Physiol. Rev..

[120] Singh A., Kukreti R., Saso L., Kukreti S. (2019). Oxidative Stress: A Key Modulator in Neurodegenerative Diseases. Molecules.

[121] Hussain T., Tan B., Yin Y., Blachier F., Tossou M.C., Rahu N. (2016). Oxidative Stress and Inflammation: What Polyphenols Can Do for Us?. Oxid. Med. Cell. Longev..

[122] Vezzani A., Friedman A., Dingledine R.J. (2013). The role of inflammation in epileptogenesis. Neuropharmacology.

[123] Lugrin J., Rosenblatt-Velin N., Parapanov R., Liaudet L. (2014). The role of oxidative stress during inflammatory processes. Biol. Chem..

[124] Li P., Chang M. (2021). Roles of PRR-Mediated Signaling Pathways in the Regulation of Oxidative Stress and Inflammatory Diseases. Int. J. Mol. Sci..

[125] Vezzani A., Balosso S., Ravizza T. (2019). Neuroinflammatory pathways as treatment targets and biomarkers in epilepsy. Nat. Rev. Neurol..

[126] Stephenson J., Nutma E., van der Valk P., Amor S. (2018). Inflammation in CNS neurodegenerative diseases. Immunology.

[127] Mishra A., Bandopadhyay R., Singh P.K., Mishra P.S., Sharma N., Khurana N. (2021). Neuroinflammation in neurological disorders: Pharmacotherapeutic targets from bench to bedside. Metab. Brain Dis..

[128] Girish C., Raj V., Arya J., Balakrishnan S. (2012). Evidence for the involvement of the monoaminergic system, but not the opioid system in the antidepressant-like activity of ellagic acid in mice. Eur. J. Pharmacol..

[129] Zeni A.L., Zomkowski A.D., Maraschin M., Rodrigues A.L., Tasca C.I. (2012). Ferulic acid exerts antidepressant-like effect in the tail suspension test in mice: Evidence for the involvement of the serotonergic system. Eur. J. Pharmacol..

[130] Nakazawa T., Yasuda T., Ueda J., Ohsawa K. (2003). Antidepressant-like effects of apigenin and 2,4,5-trimethoxycinnamic acid from Perilla frutescens in the forced swimming test. Biol. Pharm. Bull..

[131] Yi L.T., Li J.M., Li Y.C., Pan Y., Xu Q., Kong L.D. (2008). Antidepressant-like behavioral and neurochemical effects of the citrus-associated chemical apigenin. Life Sci..

[132] Machado D.G., Bettio L.E., Cunha M.P., Santos A.R., Pizzolatti M.G., Brighente I.M., Rodrigues A.L. (2008). Antidepressant-like effect of rutin isolated from the ethanolic extract from Schinus molle L. in mice: Evidence for the involvement of the serotonergic and noradrenergic systems. Eur. J. Pharmacol..

[133] Yu Y., Wang R., Chen C., Du X., Ruan L., Sun J., Li J., Zhang L., O’Donnell J.M., Pan J. (2013). Antidepressant-like effect of trans-resveratrol in chronic stress model: Behavioral and neurochemical evidences. J. Psychiatr. Res..

[134] Dhingra D., Chhillar R. (2012). Antidepressant-like activity of ellagic acid in unstressed and acute immobilization-induced stressed mice. Pharmacol. Rep..

[135] Dhapola R., Hota S.S., Sarma P., Bhattacharyya A., Medhi B., Reddy D.H. (2021). Recent advances in molecular pathways and therapeutic implications targeting neuroinflammation for Alzheimer’s disease. Inflammopharmacology.

[136] Shabab T., Khanabdali R., Moghadamtousi S.Z., Kadir H.A., Mohan G. (2017). Neuroinflammation pathways: A general review. Int. J. Neurosci..

[137] Schain M., Kreisl W.C. (2017). Neuroinflammation in Neurodegenerative Disorders—A Review. Curr. Neurol. Neurosci. Rep..

[138] Vishwakarma S., Singh S., Singh T.G. (2022). Pharmacological modulation of cytokines correlating neuroinflammatory cascades in epileptogenesis. Mol. Biol. Rep..

[139] Soltani Khaboushan A., Yazdanpanah N., Rezaei N. (2022). Neuroinflammation and Proinflammatory Cytokines in Epileptogenesis. Mol. Neurobiol..

[140] Loscher W., Potschka H., Sisodiya S.M., Vezzani A. (2020). Drug Resistance in Epilepsy: Clinical Impact, Potential Mechanisms, and New Innovative Treatment Options. Pharmacol. Rev..

[141] Brandes M.S., Gray N.E. (2020). NRF2 as a Therapeutic Target in Neurodegenerative Diseases. ASN Neuro.

[142] Liddell J.R. (2017). Are Astrocytes the Predominant Cell Type for Activation of Nrf2 in Aging and Neurodegeneration?. Antioxidants.

[143] Shahcheraghi S.H., Salemi F., Peirovi N., Ayatollahi J., Alam W., Khan H., Saso L. (2021). Nrf2 Regulation by Curcumin: Molecular Aspects for Therapeutic Prospects. Molecules.

[144] Zgorzynska E., Dziedzic B., Walczewska A. (2021). An Overview of the Nrf2/ARE Pathway and Its Role in Neurodegenerative Diseases. Int. J. Mol. Sci..

[145] Sandberg M., Patil J., D’Angelo B., Weber S.G., Mallard C. (2014). NRF2-regulation in brain health and disease: Implication of cerebral inflammation. Neuropharmacology.

[146] Osama A., Zhang J., Yao J., Yao X., Fang J. (2020). Nrf2: A dark horse in Alzheimer’s disease treatment. Ageing Res. Rev..

[147] Clements C.M., McNally R.S., Conti B.J., Mak T.W., Ting J.P. (2006). DJ-1, a cancer- and Parkinson’s disease-associated protein, stabilizes the antioxidant transcriptional master regulator Nrf2. Proc. Natl. Acad. Sci. USA.

[148] Buendia I., Michalska P., Navarro E., Gameiro I., Egea J., Leon R. (2016). Nrf2-ARE pathway: An emerging target against oxidative stress and neuroinflammation in neurodegenerative diseases. Pharmacol. Ther..

[149] Cuadrado A. (2022). Brain-Protective Mechanisms of Transcription Factor NRF2: Toward a Common Strategy for Neurodegenerative Diseases. Annu. Rev. Pharmacol. Toxicol..

[150] Morris G., Walker A.J., Walder K., Berk M., Marx W., Carvalho A.F., Maes M., Puri B.K. (2021). Increasing Nrf2 Activity as a Treatment Approach in Neuropsychiatry. Mol. Neurobiol..

[151] Brunschwig C., Leba L.J., Saout M., Martial K., Bereau D., Robinson J.C. (2016). Chemical Composition and Antioxidant Activity of *Euterpe oleracea* Roots and Leaflets. Int. J. Mol. Sci..

[152] Cardoso A.L., Pietro P.F.D., Vieira F.G.K., Boaventura B.C.B., Liz S.d., Borges G.d.S.C., Fett R., Andrade D.F.d., Silva E.L.d. (2015). Acute consumption of juçara juice (*Euterpe edulis*) and antioxidant activity in healthy individuals. J. Funct. Foods.

[153] Manolescu B.N., Oprea E., Mititelu M., Ruta L.L., Farcasanu I.C. (2019). Dietary Anthocyanins and Stroke: A Review of Pharmacokinetic and Pharmacodynamic Studies. Nutrients.

[154] Leclerc M., Dudonne S., Calon F. (2021). Can Natural Products Exert Neuroprotection without Crossing the Blood-Brain Barrier?. Int. J. Mol. Sci..

[155] Di Lorenzo C., Colombo F., Biella S., Stockley C., Restani P. (2021). Polyphenols and Human Health: The Role of Bioavailability. Nutrients.

